# Surgical Management of Pterygium Colli with Significant Skin Laxity: A Case Report

**DOI:** 10.3390/biomedicines11030984

**Published:** 2023-03-22

**Authors:** Charline Huttin, Patrick Ringenbach, Anastasia Durry, Mihai Hogas, Ionut Raducu Popescu, Alin Ciobica, Mihaela Elena Nastasa

**Affiliations:** 1 Service de Chirurgie Plastique, Reconstructive et Esthétique, CHU Hautepierre, 1 Avenue Molière, 67200 Strasbourg, France; 2 Service de Chirurgie Plastique, Reconstructive et Esthétique, GHR Mulhouse, 20 Avenue du Dr René Laennec, 68100 Mulhouse, France; 3 Physiology Department, “Grigore T. Popa” University of Medicine and Pharmacy, Universitatii 16, 700115 Iasi, Romania; 4 Department of Biology, Faculty of Biology, Alexandru Ioan Cuza University, B dul Carol I, No 11, 700115 Iasi, Romania; 5 Academy of Romanian Scientists, Splaiul Independentei Nr. 54, Sector 5, 050094 Bucuresti, Romania; 6 Center of Biomedical Research, Romanian Academy, B dul Carol I, No. 8, 700115 Iasi, Romania

**Keywords:** pterygium colli, Noonan syndrome, plastic surgery

## Abstract

Pterygium Colli or “palmate neck” is a congenital malformation that is most often part of a polimalformative syndrome. This deformity is a source of aesthetic and social embarrassment. Its correction is surgical. We present the case of a pterygium colli in a patient with Noonan syndrome. He had a significant excess of skin with posterior skin laxity, causing significant social discomfort and imposing a vicious attitude, the head bent forward. We performed a posterolateral resection of this excess by resecting two posterior triangular flaps with a resulting t-shaped scar. The results were satisfactory; the excess skin was almost completely resorbed with minimal scarring. However, this technique did not correct the low lateral hairline implantation, and there were still two lateral flaps for which the patient did not wish to have a repeat surgery.

## 1. Introduction

Pterygium colli or “webbed neck” is a congenital malformation corresponding to a “web” of skin that may extend from the mastoid to the acromion. It was first described in 1883 by Kobilinsky [1], and the term was defined by Funke [2] in 1902.

The etiology remains poorly understood. The excess skin could be the result of a cystic hygroma during embryonic life which, when regressing, would leave bilateral excess skin [3], or a growth differential leading to a skin defect in vertical dimension and a skin excess in horizontal dimension [4]. It could also sometimes be a fusion of the cervical vertebrae resulting in a neck that is too short, as in Klippel–Feil syndrome.

This malformation is often described in Turner syndrome where it is frequently accompanied by a lateral fibrous band corresponding to a thickening of the cervical fascia and a very low lateral line of capillary implantation [5]. It is also found in Klippel–Feil syndrome, Noonan syndrome, and some trisomies.

We present here our surgical management of a patient with Noonan syndrome affected by a pterygium colli with significant skin excess.

## 2. Case

**Figure 1:**
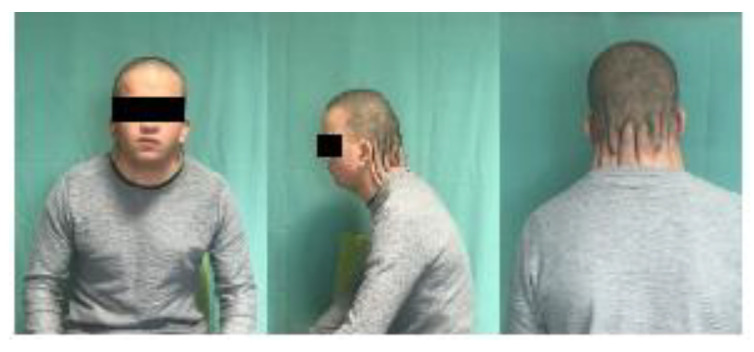
A 23-year-old man with pterygium colli associated with Noonan syndrome.

Our patient was a 23-year-old male with several congenital malformations in the context of Noonan syndrome (Figure 1). At birth he presented with cystic hygroma and severe encephalopathy, coarctation of the aorta, pterygium colli, hypertelorism with downward and outward directed palpebral slits, bilateral cryptorchidism and inner ear abnormalities, and mild mental retardation. He presented with a palmate neck with significant posterior nuqual skin excess, with skin loosening, without limitation of cervical mobility, but with a curved attitude, head bent forward. The hairline was low posteriorly. After signing the informed consent, his request was essentially aesthetic, as this malformation caused significant social embarrassment. The importance of the posterior skin excess led us to consider a posterolateral resection technique. This was also approved by the Ethical Committee of Mulhouse Hospital (No. 1 Approval of 24 November 2022).

**Figure 2:**
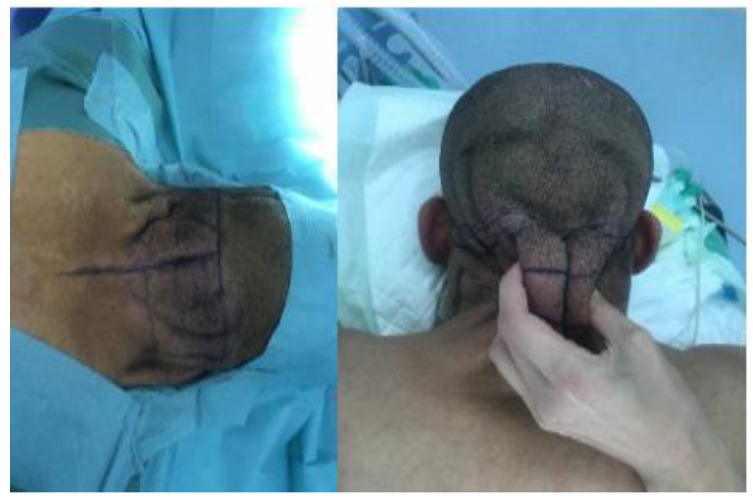
Preoperative drawing was a triangle with an upper base made with a pinch test. The result is in fact the drawing of an “A-T” flap.

The drawings were made pre-operatively, on a seated and awake patient. A horizontal mid-auricular line was drawn in the hairline area, as well as a vertical midline. Then a triangle with an upper base was drawn with a pinch test, representing the skin resection. The result is in fact the drawing of an “A-T” flap (Figure 2).

**Figure 3:**
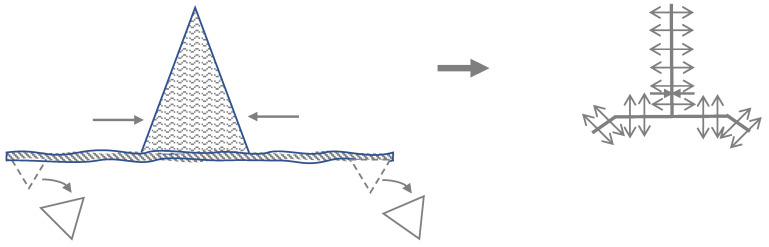
A schematic view on the A-T plasty.

Furthermore, a schematic view on the A-T plasty is presented in Figure 3.

**Figure 4:**
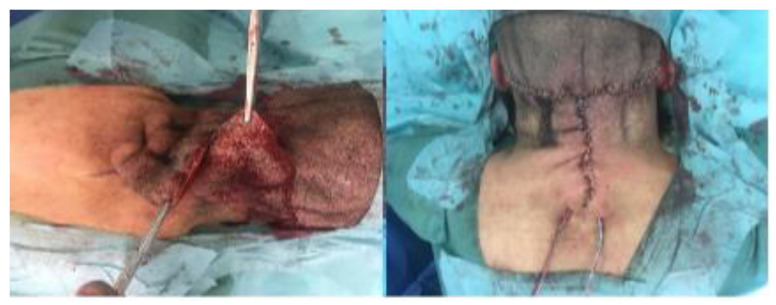
Surgical technique with the two laterals flaps peeled off in the sous cutaneous plane to the lateral border of the excess skin (intraoperative view).

Under general anesthesia, the patient was placed in the prone position. We first incised the upper horizontal line, then the vertical along the midline to the tip of the triangle. The two lateral flaps were then peeled off in the subcutaneous plane to the lateral border of the excess skin (Figure 4). Each flap was pulled to the contralateral side on either side of the midline to determine the resection. Then they were incised at this midline. The skin was sutured with skin staples with a resulting “T” shaped scar. Drainage was performed with two Blake 10 redons, removed at D1 postoperatively.

Anatomopathological analysis showed an epidermis and dermis of normal structures; some fibrotic areas were found in the hypodermis without obvious dystrophy, with ectatic vessels.

**Figure 5:**
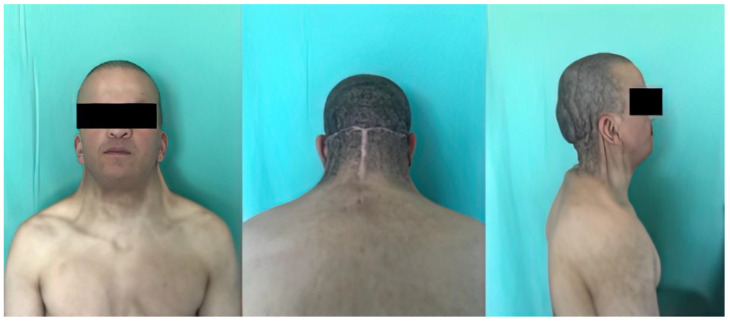
Results at 1 year postoperatively with a low hairline and lateral flanges.

In the aftermath, a small disunion occurred on the vertical scar, with a favorable evolution. Most of the excess skin was resorbed. The capillary line was only slightly raised. The scar is discreet, hidden in the hair. The patient was reviewed at 1 year, without any sign of recurrence. However, the persistence of a too low hairline and of lateral flanges were noted. For the latter, we proposed to the patient a surgical revision with lateral Z-plasties, but he refused. In fact, the patient is very satisfied with the result: he can hold his head upright and has no functional discomfort in mobilizing his neck (Figure 5).

## 3. Discussion

Noonan syndrome concerns 1/2500 births. It is due to a mutation of the PTPN11 gene, with autosomal dominant transmission. It generally includes cardiac malformations, particular facial features (poorly developed cheekbone, short nose, backward tilted ears, hypertelorism, ptosis, downward and outward eyelid slits, ogival palate, pterygium colli), short stature in adulthood, thoracic deformity, cryptorchidism, deafness, and learning difficulties. Coagulation disorders may also be present, making it necessary to systematically search for them in the preoperative workup.

Although pterygium colli is the subject of numerous publications, its surgical correction is less often discussed in the literature. Overall, posterior techniques are opposed to lateral techniques.

Initially Chandler [6] described a technique of lateral resection of the excess by Z-plasty. However, this type of scar was often hypertrophic and very visible, and did not allow correction of the line of implantation. Several authors then presented different techniques of modified lateral Z-plasties [3,7] allowing the raising of the hairline laterally, to limit the risks of scarring or recurrence by resecting the fibrous thickening of the cervical fascia.

Thompson [8] then described a technique of lateral resection in Ellipse, which could combine a resection of the upper ear according to a horizontal incision in the hair allowing to correct the low hairline implantation. Reichenberger [9] associates lateral resection in ellipse with an advancement flap for significant skin excess.

Chaput [10] more recently describes a new technique for posterior cervical lifting, by resecting the lateral skin excesses in an ellipse, and by fixing the cervical fascia posteriorly on the nuchal ligament.

Furthermore, Quian [11] performs simple lateral spindle resections for moderate skin excess, but this technique again presents a risk of scarring and does not allow correction of hair implantation.

The group of Murthy [12] presents a modified trident plasty (“M to T” plasty), where two triangular flaps are resected, with resection of the fibrous fascia. This technique allows resection of a possible fibrous flange and limits the forward displacement of the hairline, but the scars are very visible.

Posterior surgical correction techniques are less represented in the literature. Foucar [13] was the first to describe a posterior resection technique with a resulting Y-shaped scar. Schearin [14] performed a butterfly flap correction, but this technique presents numerous recurrences due to retractions.

In addition, Menick [15] provided a correction technique using lateral advancement flaps. Rossilon [16] in 1989 noted a technique with four transposition flaps, allowing correction of low and lateral hair implantation in Turner Syndrome: two lower medial flaps, transposed laterally, and two lateral and upper flaps, transposed medially. Thompson [8] in 1990 described a vertical spindle resection technique, combining, if necessary to correct hair implantation, a resection in the hair of two oblique spindles with a resulting “Y” shaped scar.

In relation to this, we can also describe here the work of Zieliński et al., which demonstrated in 2015 that the lateral approach with a shift of glabrous skin flap to the back results in an effective reduction of the webbed neck and correction of the low hairline on the side of the neck [17].

Finally, Komatsu [18] shows us a posterolateral resection technique according to a vertical spindle, associated with a horizontal resection of the hairy area, by detaching and bringing together the two lateral flaps, with a resulting “Y” shaped scar.

It is also possible to perform lateral skin expansions, in particular to expand the glabrous skin, but the morbidity of this technique and the recurrence rate explain why it has not been further developed.

The choice of these techniques depends above all on the type of pterygium colli, the excess skin which may be significant or more moderate, the need to correct the hairline, as in Turner’s syndrome, for example, where it is very often low and lateral, the need to restore neck movement if this is limited, and finally the presence of lateral fibrous bands which will have to be considered for excision or incision in order to avoid recurrence in the form of scarring. One must also take into account the high risk of keloid scars in the lateral cervical scars.

Our patient presented with a pterygium colli associated with a large posterior skin excess with a large skin slackening and a low hairline, without fibrous thickening of the cervical fascia, nor limitation of neck mobility. The request was essentially aesthetic. Our postero-lateral technique allowed us to resorb a large part of the excess, with moderate scarring, since the horizontal part of the scar was hidden in the hair.

However, this technique did not allow the removal of all the lateral excess, which was very important, nor did it correct the hairline.

As post operatory recommendations we can describe the massage of the scars at 3 weeks post surgery with a healing cream until the scars will be smooth, the application of silicone sheets or silicone cream for 2 months, as well as sun protection of the scars for 1 year.

## 4. Conclusions

The technique of posterolateral correction in TA seems to us to be adapted to a very important skin excess without lateral fibrous flanges. The resulting scars are discreet, with little risk of keloid scarring. However, this technique does not allow for correction of the hairline, nor does it allow for resection or breaking of any lateral fibrous flange.

## Data Availability

All data is available on request.
